# Comparative clinical outcomes of surgical versus conservative treatment for multiple rib fractures: A retrospective analysis

**DOI:** 10.1097/MD.0000000000043513

**Published:** 2025-08-01

**Authors:** Jin Xu, Jinnuo Fan, Liang Zhao

**Affiliations:** a Emergency Department, Wujin Hospital Affiliated to Jiangsu University, Wujin Clinical College, Xuzhou Medical University, Changzhou, Jiangsu Province, China.

**Keywords:** chest trauma, multiple rib fracture, non-flail chest, quality of life, surgical treatment

## Abstract

This study explores the clinical effects of surgical treating and conservative treating for multiple rib fractures (MRF). The study selected patients with MRF admitted to the thoracic surgery and emergency department of our hospital from 2018 to 2023 as the research subjects. They were separated into 2 groups according to different treatment methods: one received surgical treating, and the other one received conservative treating. A questionnaire survey was conducted to evaluate the psychological health, postoperative recovery, comorbidities, and lung function recovery of the patients. During hospitalization, there were 23 patients with complications in the surgical group. There were 11 cases of pleural effusion, 3 of delayed hemothorax, 6 of pulmonary infection, 2 of atelectasis, and 1 of rib displacement at the fractured end. Compared to the conservative treatment group, patients with pulmonary infections were much smaller (*P* < .05). On the 2nd and 4th day after treatment, the pH values of patients in the surgical group were 7.37 ± 0.04 and 7.41 ± 0.03, respectively. The blood pH values of patients in the conservative treatment group were 7.33 ± 0.05 and 7.43 ± 0.06, respectively. The blood oxygen partial pressure values of the surgical group patients were 89.77 ± 10.41 mm Hg and 90.02 ± 9.74 mm Hg, respectively. The blood oxygen partial pressure values of the conservative treatment group were 77.47 ± 15.63 mm Hg and 82.57 ± 10.51 mm Hg, respectively. Two groups’ data comparison showed *P* < .05. For the treatment of MRF in non-flail chest, surgical treatment can reduce the incidence of pulmonary infection. Meanwhile, surgical treatment can shorten the medication time and significantly improve the quality of life of patients after treatment.

## 1. Introduction

Chest trauma accounts for approximately 13.5% of all injuries, with a primary cause often being external violence. This trauma significantly impacts patient health and is a leading cause of mortality.^[1]^ Multiple rib fractures (MRF), which typically result from high-energy trauma such as car accidents, falls, or sports injuries, are common in chest trauma cases. As the ribs play a crucial role in protecting vital internal organs, MRF can lead to severe physiological and functional damage.^[2,3]^ The management of MRF is often complex, with treatment approaches varying depending on whether the injury is classified as flail chest or non-flail chest. While flail chest MRF typically requires surgical intervention due to the instability it causes in the chest wall,^[4]^ non-flail chest MRF (despite being less severe in terms of chest wall instability) still presents significant challenges, including respiratory complications, pain management, and prolonged recovery.

However, there remains limited evidence comparing surgical versus conservative treatments for non-flail chest MRF.^[5]^ Though both approaches aim to alleviate pain, support rib stability, and promote lung function recovery, it is unclear whether one method offers significant clinical advantages over the other, especially regarding complications and long-term outcomes. Therefore, this study aims to evaluate the clinical effects of surgical treatment versus conservative treatment for non-flail chest MRF, focusing on factors such as postoperative recovery, complications, pain management, and psychological health. By addressing this gap in the existing literature, we aim to provide evidence that can guide clinical decision-making for non-flail chest MRF patients. The primary outcome of the study was to assess the incidence of lung infections that occurred during treatment in both groups. Lung infection is a common complication of MRF and is strongly associated with delayed recovery and high morbidity in patients. Secondary outcomes included a comparison of the duration of drug use (such as pain medication and antibiotic use) between the surgical and conservative treatment groups. The recovery of lung function in the 2 groups was evaluated by blood gas analysis and other pulmonary function measures. Patients’ quality of life after treatment was assessed using standard quality of life measures and evaluated at follow-up. The incidence of other complications (such as pleural effusion, delayed hemothorax, atelectasis, rib displacement, etc) during hospitalization was compared between the 2 groups.

## 2. Objects and methods

### 2.1. Research objects

This study was approved by the Ethics Committee of Wujin Hospital Affiliated to Jiangsu University. MRF patients admitted to the thoracic surgery and emergency department of our hospital from 2018 to 2023 were selected as the research subjects. Inclusion criteria: patients with abbreviated injury scale (AIS) of <4 in the chest; age ≥ 18 years old; diagnosed fracture in patients with rib count >2 through three-dimensional reconstruction of chest ribs; no history of chronic diseases such as hypertension, diabetes, liver, and kidney dysfunction in the past; rib fractures may or may not be accompanied by displacement and significant pain on the injured side; informed consent and voluntary participation in this study. Exclusion criteria: patients with combined traumatic brain injury and head AIS >3; patients with severe abdominal injuries and abdominal AIS >3; having cognitive and expression impairments, postoperative follow-up is not possible; patients diagnosed with flail chest, abnormal breathing, and chest collapse. Two hundred seventy-four MRF patients were admitted from 2018 to 2023, and 124 met the inclusion criteria. The surgical group had 61 cases. The conservative group had 63 cases. All included patients were followed up with informed consent.

### 2.2. Treatment methods

#### 2.2.1. Surgical procedure

Following successful induction of general anesthesia, double-lumen endotracheal intubation was performed, and the patient was positioned in the lateral decubitus position. The surgical field was prepared using a standardized three-stage iodine-based antisepsis protocol prior to sterile draping. Surgical incision planning integrated preoperative chest CT data with three-dimensional rib fracture reconstructions, supplemented by intraoperative physical examination findings and patient positioning considerations. This multimodal approach aimed to optimize incision selection by balancing minimally invasive principles, intraoperative accessibility, and postoperative cosmetic outcomes.^[6]^

A thoracic wall incision was made through skin and subcutaneous tissues, followed by layered dissection of muscular planes with attention to fiber orientation to minimize iatrogenic muscle injury. Intercostal muscle division along the rib axis permitted exposure of fracture sites, maintaining approximately 3 cm of rib mobilization on either side of the fracture line. Meticulous subperiosteal dissection was avoided to preserve rib vascularity, with particular care taken to protect neurovascular bundles during deep tissue mobilization. Patients presenting with intact pleura, hemopneumothorax, or pulmonary contusions underwent extrapleural external fixation to maintain thoracic integrity and reduce postoperative complications.

Titanium rib fixation plates (Waston Medical) were selected based on quantitative measurements of rib morphology including length, width, and curvature. Implant preparation involved precooling in sterile 0 to 4 °C saline to enhance malleability. Anatomic contouring was performed using dedicated rib-shaping instruments when necessary. Fixation arms were opened to 120% of measured rib width and centered over fracture lines, ensuring bilateral placement of 2 fixation arms per fracture segment. Sequential clamping commenced with terminal fixation points followed by intermediate arms, applying single-application force to achieve stable fixation without metal fatigue. Intraoperative cooling with chilled saline was utilized for plate repositioning when required.

In polytrauma cases with bilateral rib fractures, unilateral fixation preceded contralateral intervention. Concomitant life-threatening injuries received priority management per Advanced Trauma Life Support protocols. Hemostasis was achieved through systematic hematoma evacuation and electrocautery prior to layered wound closure. Routine postoperative management included thoracostomy drainage (24–48 hours), prophylactic cephalosporin administration (cefazolin 1g q8h), multimodal analgesia, and early ambulation protocols. Chest radiographs were obtained at 72-hour intervals to monitor fixation integrity and guide drain removal decisions.

#### 2.2.2. Nonsurgical treatment

Following admission, standardized laboratory evaluations including complete blood count, serum biochemistry, arterial blood gas analysis, and electrolyte profiling were systematically conducted. Supplemental oxygen therapy (2–5 L/min via nasal cannula) was administered to optimize pulmonary function, coupled with controlled crystalloid infusion (20–30 mL/kg/24 h) to maintain peripheral perfusion and mitigate thromboembolic risks. Hemodynamic stabilization protocols addressed shock states, acid-base imbalances, and electrolyte disturbances through targeted interventions aligned with Advanced Trauma Life Support guidelines. Thoracic stabilization commenced with CT-guided identification of fracture sites (1 mm slice thickness), followed by sequential application of pressure-distributing cotton padding (5 cm thickness) and elastic rib fixation belts (Medi® Dynacor, Medtronic plc, Dublin, Ireland) to restrict chest wall mobility (<2 cm respiratory displacement). Pulmonary hygiene protocols incorporated mechanized secretion clearance (MetaNeb® System, Hillrom/Baxter International Inc., Chicago), scheduled postural drainage, and incentive spirometry training. Analgesic management employed scheduled NSAIDs (ketorolac 30 mg IV q6h) with opioid rescue dosing (hydromorphone 0.2 mg IV PRN). Antimicrobial prophylaxis (cefuroxime 1.5 g q8h) and closed thoracic drainage were instituted for hemopneumothorax cases, while methylprednisolone (1 mg/kg/day) was reserved for radiologically confirmed pulmonary contusions with inflammatory exudates. Mechanical ventilation (Hamilton C6) was initiated for respiratory failure (PaO₂/FiO₂ <200 mm Hg), with tracheostomy (Blue Rhino® Percutaneous Kit, Cook Medical LLC, Bloomington) performed when prolonged intubation (>7 days) was anticipated. For suboptimal belt fixation, nano-polymer chest guards (T-Lock®, DePuy Synthes, Johnson & Johnson, Raynham) were applied following CT-confirmed fracture localization: The target area was prepped with 70% isopropanol over a 15 × 15 cm field, after which vacuum-sealed splints were thermally activated in 70 ± 2 °C water (120 seconds), air-cooled to 40 °C, and anatomically positioned during coached maximal inspiration, secured with medical-grade adhesive tape at interface gaps.

### 2.3. Observation indicators

#### 2.3.1. General information comparison

This included the patient’s age, gender, number of rib fractures, ISS score, and AIS score.

#### 2.3.2. Treatment evaluation

Serial arterial blood gas analyses were performed at 3 clinical timepoints: upon admission, 48 hours post-intervention, and 96 hours post-intervention, with key parameters including pH (7.35–7.45), PaO₂ (80–100 mm Hg), and SaO₂ (95–100%) being systematically measured. Pain intensity was quantified using the visual analog scale (VAS),^[7,8]^ with scores stratified as follows: 7 to 10 (severe discomfort requiring immediate pharmacological intervention), 4 to 6 (moderate pain interfering with sleep architecture), ≤3 (mild tolerable sensation), and 0 (asymptomatic status). Analgesic consumption patterns were documented through medication administration records, including frequency (doses/24h) and temporal parameters (hours between administrations). Clinical outcomes monitoring encompassed: (1) incidence of clinically significant complications (pneumonia/ARDS rates), (2) healthcare utilization metrics (total length of stay in days), (3) pharmacoeconomic data (total hospitalization costs in USD), and (4) antimicrobial stewardship indicators (duration of antibiotic therapy in days).^[9]^

#### 2.3.3. Evaluation of postoperative follow-up indicators

Postoperative outcomes were assessed through scheduled outpatient follow-ups conducted at 2 to 5 months (median 3-month) intervals. Concurrent pain evaluation employed dynamic VAS measurements during standardized maneuvers (deep breathing/coughing), with scores stratified as: 0 (asymptomatic), 1 to 3 (mild/no functional limitation), 4 to 6 (moderate/night disturbance), and 7 to 10 (severe/activity-restricting).

### 2.4. Statistical methods

A database was established using SPSS 22.0 statistical software, with survey data entered separately by 2 individuals and then checked by a third person. When inconsistent data appeared, the data was checked and verified to be correct before conducting statistical analysis. Count data are expressed in%, while measurement data are expressed in mean plus error, and *T* test is used. *P* < .05 indicates that the difference is statistically significant. A power analysis was conducted prior to the study, and a sample size of 124 patients (61 in the surgical group and 63 in the conservative group) was determined to provide 80% power to detect a clinically significant difference in the incidence of pulmonary infections between the 2 groups, at a significance level of 0.05. The sample size calculation was based on previous studies that suggested a potential difference in pulmonary infection rates and other secondary outcomes.

### 2.5. Quality control

After obtaining ethical approval and informed consent, a formal questionnaire survey was conducted. All investigators received standardized training to ensure a thorough understanding of the study’s objectives and procedures. During the survey process, consistent instructional language was used to minimize interviewer bias.^[10,11]^ The inclusion and exclusion criteria were strictly defined, and participant confidentiality was maintained throughout the study. Completed questionnaires were collected immediately and checked item by item for completeness. During data entry and analysis, responses were double-checked for accuracy and consistency, and logical checks were performed to detect discrepancies. Questionnaires exhibiting obvious logical errors or with >25% of items unanswered were excluded from the analysis.

A questionnaire was considered invalid under the following conditions: if responses showed a patterned or uniform trend (e.g., wavy or linear filling); if more than half of the items were left unanswered; or if >25% of the responses were repeated. In cases where repeated answers accounted for <25%, only the duplicated items were removed, and the remaining data were retained for analysis.^[12,13]^

### 2.6. Ethical considerations and informed consent

The study protocol was approved by the Ethics Committee of Wujin Hospital affiliated with Jiangsu University. All participants were informed that the study would not interfere with their ongoing medical treatment and that their participation was entirely voluntary. Informed consent was obtained prior to enrollment. To protect privacy, all patient data were anonymized using unique identification codes, and no personal identifiers were disclosed at any stage of the study or in the dissemination of the results.

## 3. Results

### 3.1. Comparison of patients’ basic information

One hundred twenty-four patients were included and divided into a surgical group of 61 cases and a conservative treatment group of 63 cases according to the treatment method in Table 1. The surgical group had 47 male and 14 female subjects, averaging (58.92 ± 20.04) years old, an average body mass index (BMI) of 23.67 ± 1.74, and an average fracture rib of 4.10 ± 1.20. The conservative treatment group had 46 male and 17 female subjects, averaging (55.77 ± 19.36) years old. The mean BMI was 23.87 ± 1.67, and the mean number of fractured ribs was 4.31 ± 1.17. These basic data comparisons showed *P* > .05.

**Table 1:** Comparison of patients’ basic information.

Clinical features	Surgical group (n = 61)	Nonsurgical group (n = 63)	*t*	*P*
Gender (man/woman)	47/14	46/17	0.127	.799
Age	58.92 ± 20.04	55.77 ± 19.36	0.681	.556
BMI	23.67 ± 1.74	23.87 ± 1.67	1.358	.224
Number of fractured ribs	4.10 ± 1.20	4.31 ± 1.17	1.404	.209
AIS	2.39 ± 0.47	2.21 ± 0.41	0.476	.696
ISS	9.14 ± 2.32	9.19 ± 2.47	0.474	.698

### 3.2. Analysis of injury causes and comparison of fractures

The number of rib fractures in 124 patients was analyzed in Table 2. In the surgical group, there were more patients who broke 3 ribs and 6 or more ribs, and fewer patients who broke 5 ribs. In the conservative treatment group, there were 23 patients with 3 broken ribs, accounting for 36.5%. There were 6 patients with 5 broken ribs, accounting for 9.5%. Two groups’ fractured ribs comparison showed *P* > .05.

**Table 2:** Comparison of fractures in patients.

Number of rib fractures	Surgical group (n = 61)	Nonsurgical group (n = 63)	*t*	*P*
3	21 (34.4)	23 (36.5)	6.757	.097
4	14 (22.9)	13 (20.6)	/	/
5	3 (4.9)	6 (9.5)	/	/
6	7 (11.4)	7 (11.1)	/	/
>6	16 (26.2)	14 (22.2)	/	/

**Figure 1.**
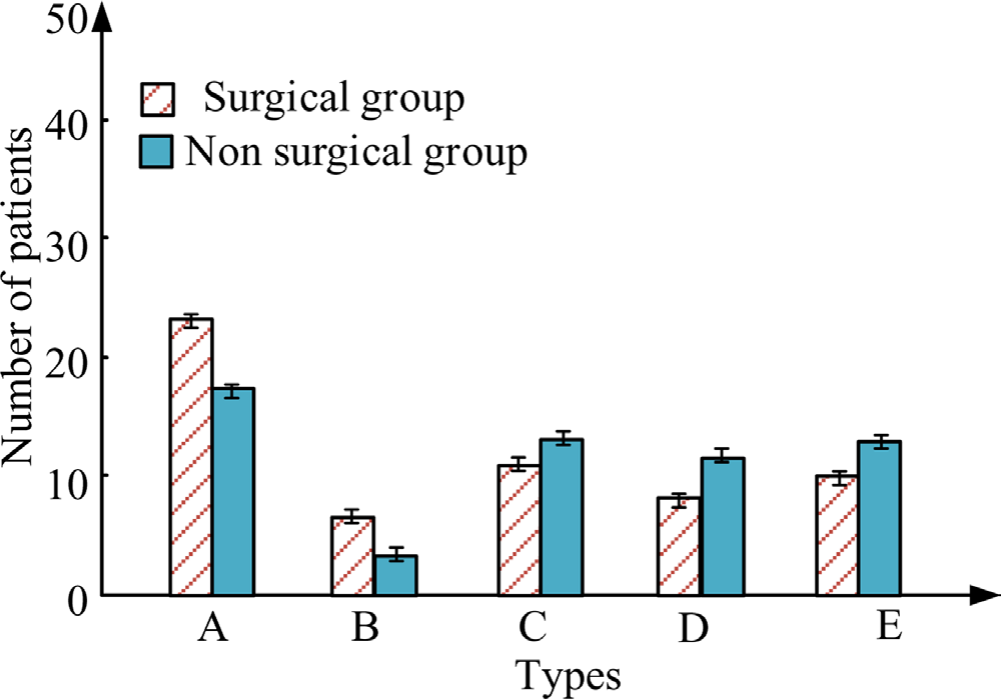
Results of injury cause.

An analysis was conducted on the causes of injuries in 124 patients, as shown in Fig. 1. A–E represents road traffic injury, high-altitude falling injury, crushing and collision injury, heavy object impact injury, and mechanical accident injury, respectively. In these 2 groups, fractures were mainly caused by traffic accidents, followed by compression collisions. In the surgical group, patients with fractures caused by heavy object impact were the lowest. The comparison results of these 2 groups showed *P* > .05.

### 3.3. Comparison of complications during hospitalization

The incidence rate of complications of the 2 groups during hospitalization was compared in Table 3. During hospitalization, the surgical group had 23 patients with complications, including 11 (18.0%) of pleural effusion, 3 (4.9%) of delayed hemothorax, 6 (9.8%) of pulmonary infection, 2 (3.3%) of atelectasis, and 1 (1.6%) of rib displacement at the severed end. The surgical group had smaller pulmonary infections than the conservative treatment group (*P* < .05). All other comparisons showed *P* > .05.

**Table 3:** Comparison of complications during hospitalization of patients.

Related complications	Surgical group (n = 61)	Nonsurgical group (n = 63)	*t*	*P*	OR	Effect size
Pleural effusion	11 (18.0)	13 (20.6)	0.401	.514	0.85 (95% CI: 0.31–2.31)	0.10
Delayed hemopneumothorax	3 (4.9)	2 (3.1)	0.007	.942	1.61 (95% CI: 0.23–11.18)	0.06
Pulmonary infection rate	6 (9.8)	14 (21.6)	4.621	.011	0.39 (95% CI: 0.15–1.02)	0.67
Atelectasis rate	2 (3.3)	4 (6.3)	0.074	.808	0.51 (95% CI: 0.08–3.29)	0.14
Displacement of fractured rib	1 (1.6)	2 (3.2)	2.939	.068	0.47 (95% CI: 0.05–4.08)	0.10

### 3.4. Patient blood gas analysis

The blood gases of 2 groups before admission were analyzed on the 2nd and 4th day after treatment in Table 4. The comparison of pH, PaO_2_, PaCO_2_, and PaO_2_/FiO_2_ before treatment between these 2 groups showed *P* > .05. On the 2nd and 4th day after treatment, the pH of the surgical group was 7.37 ± 0.04 and 7.41 ± 0.03, respectively, while the pH of the conservative treatment group was 7.33 ± 0.05 and 7.43 ± 0.06 (*P* < .05). The PaO_2_ levels of patients in the surgical group were 89.77 ± 10.41 mm Hg and 90.02 ± 9.74 mm Hg, respectively, while the PaO_2_ levels in the conservative treatment group were 77.47 ± 15.63 mm Hg and 82.57 ± 10.51 mm Hg, respectively (*P* < .05). The PaCO_2_ levels of patients in the surgical group were 43.07 ± 10.31 mm Hg and 40.76 ± 9.69 mm Hg, respectively, while those in the conservative treatment group were 47.62 ± 12.97 mm Hg and 46.97 ± 11.32 mm Hg, respectively (*P* < .05). Therefore, this surgical group had better recovery than this conservative treatment group.

**Table 4:** Patients’ blood gas analysis.

Project	Surgical group (n = 61)	Nonsurgical group (n = 63)	*t*	*P*	Effect size (Cohen *d*)
pH					
Admission	7.02 ± 0.17	6.98 ± 0.24	1.867	.074	0.23
Day 2	7.37 ± 0.04	7.33 ± 0.05	‐3.113	.002	0.91
Day 4	7.41 ± 0.03	7.43 ± 0.06	‐2.476	<.001	‐0.44
PaO_2_ (mm Hg)					
Admission	62.14 ± 9.21	62.71 ± 7.33	‐0.956	.264	0.05
Day 2	89.77 ± 10.41	77.47 ± 15.63	‐4.657	<.001	0.99
Day 4	90.02 ± 9.74	82.57 ± 10.51	‐5.398	<.001	0.73
PaCO_2_ (mm Hg)					
Admission	53.62 ± 8.71	53.87 ± 9.47	‐0.962	.271	0.02
Day 2	43.07 ± 10.31	47.62 ± 12.97	3.676	<.001	‐0.40
Day 4	40.76 ± 9.69	46.97 ± 11.32	4.264	<.001	‐0.60
PaO_2_/FiO_2_					
Admission	196.43 ± 42.87	199.33 ± 59.52	0.762	.368	0.08
Day 2	342.67 ± 58.74	252.66 ± 51.54	‐8.487	<.001	1.62
Day 4	391.22 ± 71.67	321.67 ± 65.77	‐6.774	<.001	1.01

PaO_2_ refers to blood oxygen partial pressure, PaCO_2_ refers to blood carbon dioxide partial pressure, and FiO_2_ refers to the oxygen concentration fraction in the inhaled gas.

### 3.5. Comparison of pain scores

The pain scores of these 2 groups were compared in Table 5. At admission, the VAS of patients in 2 groups were 6.72 ± 2.21 and 7.11 ± 1.68, respectively (*P* > .05). After 2 and 4 days of hospitalization, the VAS scores of the surgical group were 3.68 ± 0.88 and 1.67 ± 0.68, respectively, while the VAS scores of the conservative treatment group were 2.76 ± 0.84 and 1.36 ± 0.39, respectively. The VAS comparison between these 2 groups showed *P* < .05.

**Table 5:** Patients’ pain scores.

Assessment	Surgical group (n = 61)	Nonsurgical group (n = 63)	*t*	*P*	Effect size
VAS score upon admission	6.72 ± 2.21	7.11 ± 1.68	‐1.498	.247	‐0.20
VAS score after 2 days of treatment	3.68 ± 0.88	2.76 ± 0.84	6.539	<.001	1.07
VAS score after 4 days of treatment	1.67 ± 0.68	1.36 ± 0.39	7.639	<.001	0.53

### 3.6. Analysis of mental health status

The psychological health status of these 2 groups after treatment was analyzed in Table 6. In the HAMA score, 2 groups’ psychological state scores before treatment were 51.78 ± 4.57 and 52.71 ± 4.87, respectively, and the scores after treatment were 21.64 ± 3.36 and 32.36 ± 5.41 (*P* < .05). In the HAMD score, the psychological state scores of the surgical group and the conservative treatment group before treatment were 52.66 ± 4.37 and 50.87 ± 5.14, respectively, and the scores after treatment were 28.74 ± 4.14 and 38.62 ± 4.37 (*P* < .05). Therefore, the psychological recovery of patients after surgery was not inferior to that of patients in the other group.

**Table 6:** Analysis of mental health status.

Group	N	HAMA rating		HAMD rating	
		Before operation	After operation	Before operation	After operation
Surgical group	61	51.78 ± 4.57	21.64 ± 3.36	52.66 ± 4.37	28.74 ± 4.14
Nonsurgical group	63	52.71 ± 4.87	32.36 ± 5.41	50.87 ± 5.14	38.62 ± 4.37
*t*		‐0.67	11.94	‐0.47	12.56
*P*		0.46	0.01	0.57	0.01
Effect size		‐0.20	‐2.56	0.38	‐2.32

HAMA refers to the Hamilton Anxiety Scale, and HAMD refers to the Hamilton Depression Scale.

### 3.7. Comparison of pulmonary function recovery

**Figure 2.**
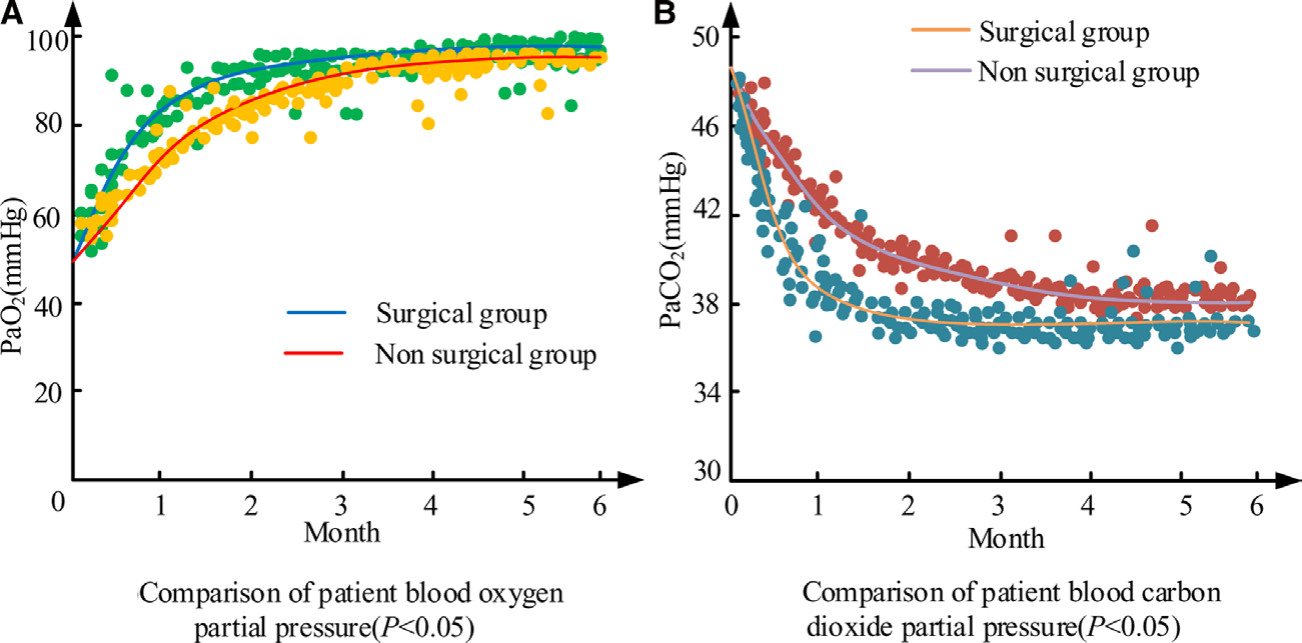
Recovery of lung function.

Due to the impact of rib fracture on lung function, in Fig. 2, the recovery of lung function in patients was compared. Figure 2A shows the blood oxygen partial pressure of patients in 2 groups after treatment. Figure 2B shows the post treatment blood carbon dioxide partial pressure of patients in 2 groups. In Fig. 2A, both groups experienced mild hypoxia after treatment. After treatment, the blood oxygen partial pressure of surgical patients quickly returned to normal levels, and compared to the conservative treatment group, their recovery speed was faster. In Fig. 2B, both groups showed elevated blood carbon dioxide partial pressure after treatment. After treatment, both groups showed a decrease in blood carbon dioxide partial pressure. The decrease in blood carbon dioxide partial pressure in the surgical group was faster and more significant.

### 3.8. Efficacy evaluation

The treatment effects of 2 groups were analyzed in Table 7. The surgical group had longer hospitalization time than the other group (*P* < .05). The duration of analgesic use in the surgical group and conservative treatment group was 4.34 ± 1.26 days and 5.64 ± 1.84 days, respectively. The surgical group had better fracture recovery and lower postoperative pain score than the conservative treatment group (*P* < .05).

**Table 7:** Efficacy analysis.

Evaluating indicator	Surgical group (n = 61)	Nonsurgical group (n = 63)	*t*	*P*	Effect size
Hospital stay(d)	21.32 ± 2.56	18.74 ± 3.62	4.725	<.001	0.84
Analgesics usage time(d)	4.34 ± 1.26	5.64 ± 1.84	14.249	<.001	0.88
Degree of fracture healing	/	/	6.754	<.001	0.63
Good	49	46	/	/	
Acceptable	11	9	/	/	
Ordinary	1	8	/	/	
VAS	/	/	2.542	.006	0.36
Painless	47 (77.1)	42 (66.7)	/	/	
Mild pain	10 (16.4)	13 (20.6)	/	/	
Moderate pain	4 (6.6)	8 (12.7)	/	/	
Severe pain	0 (0)	0 (0)	/	/	

### 3.9. Quality of life after treatment

**Figure 3.**
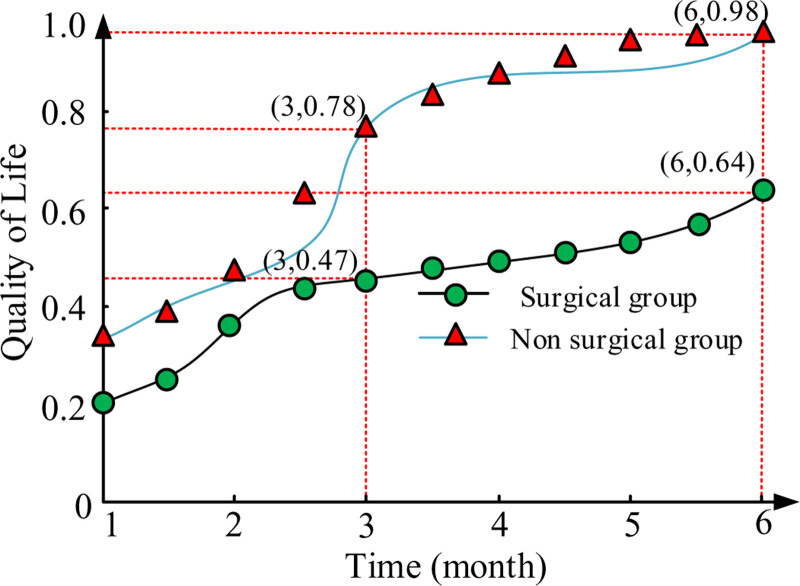
Postoperative quality of life.

After treatment, these 2 groups were followed up for 6 months. Figure 3 shows the quality of life of the patients in both groups. After 3 months of treatment, the quality of life of these 2 groups was 0.78 and 0.47, respectively. After 6 months, their quality of life was 0.98 and 0.64, respectively. After surgical treatment, the quality of life of patients has significantly improved.

## 4. Discussion

While the country is vigorously developing infrastructure, traffic accidents are becoming more frequent, and the chest injuries caused by traffic are significantly increasing. MRF refers to the situation where multiple ribs in the chest are simultaneously or continuously fractured, which usually occurs in severe trauma or accidents.^[14,15]^ MRF may cause severe chest pain, breathing difficulties, lung injury, and other complications. The common types of MRF are flail chest MRF and non-flail chest MRF. Patients with flail chest MRF who undergo surgical treatment have better prognosis than those who do not.^[16]^ Non-flail chest MRF refers to MRF in the chest, but does not cause detachment or collapse of the chest in the injured area. Although severe chest pain and breathing difficulties may also occur, the stability of the chest has not been significantly affected. Therefore, the clinical efficacy of surgical and conservative treatment for non-flail chest MRF is not clear.

This study selected MRF patients admitted to the thoracic surgery and emergency department of our hospital from 2018 to 2023 as the research subjects. They were separated into 2 groups, one receiving surgical treatment and the other receiving conservative treatment. The psychological health, postoperative recovery, complications, and lung function recovery of patients were evaluated through a questionnaire survey. One hundred twenty-four patients were included and divided into surgical group (61 cases) and conservative treatment group (63 cases) based on treatment methods. The surgical group had 47 male and 14 female subjects, averaging (58.92 ± 20.04) years, a mean BMI of 23.67 ± 1.74, and a mean fracture rib of 4.10 ± 1.20. The conservative treatment group had 46 male and 17 female subjects, averaging (55.77 ± 19.36) years old. The mean BMI was 23.87 ± 1.67, and the mean fractured rib was 4.31 ± 1.17. In the surgical group, there were more patients who broke 3 ribs and 6 or more ribs, and fewer patients who broke 5 ribs. In the conservative treatment group, there were 23 patients with 3 broken ribs, accounting for 36.5%. There were 6 patients with 5 broken ribs, accounting for 9.5%. The incidence rate of complications during hospitalization was compared between these 2 groups. During hospitalization, there were a total of 23 patients with complications in the surgical group. There were 11 cases of pleural effusion, 3 of delayed hemothorax, 6 of pulmonary infection, 2 of atelectasis, and 1 of rib displacement at the fractured end. The pulmonary infections were much smaller than the conservative treatment group, which was similar to the results of Lucinda A et al.^[17]^ The blood gas of these 2 groups before admission was analyzed on the 2nd and 4th day after treatment. The comparison of pH, PaO_2_, PaCO_2_, and PaO_2_/FiO_2_ before treatment showed *P* > .05. On the 2nd and 4th day after treatment, the pH values of patients in the surgical group were 7.37 ± 0.04 and 7.41 ± 0.03, respectively, while those in the conservative treatment group were 7.33 ± 0.05 and 7.43 ± 0.06, respectively. The PaO_2_ of the surgical group patients were 89.77 ± 10.41 mm Hg and 90.02 ± 9.74 mm Hg, respectively, while the PaO_2_ of the conservative treatment group was 77.47 ± 15.63 mm Hg and 82.57 ± 10.51 mm Hg, respectively. The PaCO_2_ levels of patients in the surgical group were 43.07 ± 10.31 mm Hg and 40.76 ± 9.69 mm Hg, respectively, while those in the conservative treatment group were 47.62 ± 12.97 mm Hg and 46.97 ± 11.32 mm Hg, respectively. Comparing the pain scores of 2 groups of patients, the VAS of the surgical group and the conservative treatment group at admission were 6.72 ± 2.21 and 7.11 ± 1.68, respectively. On the 2nd and 4th day of hospitalization, the VAS scores of the surgical group were 3.68 ± 0.88 and 1.67 ± 0.68, respectively, while the VAS scores of the conservative treatment group were 2.76 ± 0.84 and 1.36 ± 0.39, respectively. Both groups of patients experienced mild hypoxia after treatment. After treatment, the blood oxygen partial pressure of surgical patients quickly returned to normal levels, and compared to the conservative treatment group, their recovery speed was faster. The psychological health status of these 2 groups after treatment was analyzed. In the HAMA score, the psychological state scores of these 2 groups before treatment were 51.78 ± 4.57 and 52.71 ± 4.87, respectively. The posttreatment scores were 21.64 ± 3.36 and 32.36 ± 5.41, respectively (*P < *.05). In the HAMD score, the psychological state scores of the surgical group and the conservative treatment group before treatment were 52.66 ± 4.37 and 50.87 ± 5.14, respectively. The posttreatment scores were 28.74 ± 4.14 and 38.62 ± 4.37, respectively, which was similar to the research results of Bhattacharya D et al.^[18]^ An analysis was conducted on the therapeutic effects of these 2 groups. The surgical group had longer hospital stay than the conservative treatment group leader. The duration of analgesic use in 2 groups was 4.34 ± 1.26 days and 5.64 ± 1.84 days, respectively. The surgical group had better fracture recovery and lower postoperative pain scores than the conservative treatment group. The mechanisms underlying these improved outcomes in the surgical group can be explained through several factors: 1. surgical fixation of the ribs provides better chest wall stability, which directly contributes to more effective ventilation and respiratory mechanics. Stabilizing the fractures reduces the mechanical strain on the chest wall, allowing for more efficient lung expansion and better oxygenation. This likely explains the significant improvement in pulmonary function observed in the surgical group, as evidenced by the higher PaO_2_ levels and quicker recovery from hypoxia. 2. One of the most notable findings of this study was the significantly lower rate of pulmonary infections in the surgical group (9.8% vs 21.6%, *P* < .05). Surgical stabilization may help reduce complications such as atelectasis, pneumothorax, and pulmonary contusions, which are often associated with inadequate chest wall stabilization. By preventing rib displacement, surgery likely minimizes the risk of lung contusion and atelectasis, which can contribute to secondary infections. Furthermore, the early mobilization facilitated by surgical stabilization may help to prevent postoperative pneumonia, a common complication in chest trauma patients. 3. Surgical stabilization likely plays a role in reducing chronic pain, which is a significant concern in MRF patients. Stabilizing displaced ribs can help reduce pain associated with rib movement, making it easier for patients to engage in deep breathing exercises and rehabilitation. This, in turn, may have contributed to the observed improvement in psychological health scores in the surgical group, as patients report less pain and greater comfort.

As a retrospective observational study, patient selection could introduce bias. Although we matched patients based on inclusion criteria, factors such as injury severity and patient preferences may have influenced the decision to proceed with surgery. It is possible that more severe cases were preferentially treated surgically, which could contribute to differences in hospital stay length, complications, and recovery times. Future prospective randomized controlled trials would help to minimize such biases and provide more robust evidence.

The retrospective design inherently limits the ability to establish causal relationships between treatment and outcomes. We relied on historical data, which means that unmeasured confounders or changes in clinical practice over time could have influenced results. Additionally, missing data and variations in follow-up practices may have affected the consistency of the findings. While our study offers important insights into the treatment of non-flail chest MRF, its applicability to other populations may be limited. The study was conducted at a single institution with a specific patient demographic, and findings may not fully represent younger populations, different geographic regions, or patients with varying healthcare access. The results might not be directly translatable to pediatric patients or those with more chronic comorbidities (e.g., cardiovascular disease, diabetes) that might influence treatment outcomes. Therefore, multicenter studies and population-based analyses are needed to further validate these findings and assess their generalizability.

In conclusion, our study demonstrates that surgical treatment for non-flail chest MRF significantly reduces complications (especially pulmonary infections), enhances lung function recovery, and improves pain management and psychological health compared to conservative treatment. Furthermore, surgical treatment leads to better long-term quality of life for patients with MRF. These findings suggest that surgical intervention should be considered as a viable option for patients with non-flail chest MRF, particularly when aiming to improve patient outcomes and reduce complications.

## Author contributions

**Conceptualization:** Jin Xu, Liang Zhao.

**Data curation:** Jin Xu, Jinnuo Fan, Liang Zhao.

**Formal analysis:** Jin Xu, Jinnuo Fan, Liang Zhao.

**Investigation:** Jin Xu, Jinnuo Fan, Liang Zhao.

**Methodology:** Jin Xu, Jinnuo Fan, Liang Zhao.

**Supervision:** Jinnuo Fan.

**Writing – original draft:** Jin Xu, Liang Zhao.

**Writing – review & editing:** Jin Xu, Liang Zhao.
